# Mitochondrial Quality Control Orchestrates the Symphony of B Cells and Plays Critical Roles in B Cell-Related Diseases

**DOI:** 10.1155/2024/5577506

**Published:** 2024-10-17

**Authors:** Wuhao Li, Peiyang Cai, Ye Xu, Weihong Tian, Licong Jing, Qiaoyi Lv, Yangjing Zhao, Hui Wang, Qixiang Shao

**Affiliations:** ^1^Jiangsu Key Laboratory of Medical Science and Laboratory Medicine, School of Medicine, Jiangsu University, Zhenjiang 212013, Jiangsu, China; ^2^Institute of Medical Genetics and Reproductive Immunity, The Digestive and Reproductive System Cancers Precise Prevention Engineering Research Center of Jiangsu Province, Jiangsu College of Nursing, Huai'an 223002, Jiangsu, China

**Keywords:** B cell, B cell-related diseases, mitochondria, mitochondrial dynamics, mitochondrial quality control

## Abstract

B cells are essential for humoral immune response due to their ability to secrete antibodies. The development of B cells from the bone marrow to the periphery is tightly regulated by a complex set of immune signals, and each subset of B cells has a unique metabolic profile. Mitochondria, which serve as cellular energy powerhouses, play an essential role in regulating cell survival and immune responses. To maintain metabolic homeostasis, mitochondria dynamically adjust their morphology, distribution, and mass via biogenesis, fusion and fission, translocation, and mitophagy. Despite its extreme importance, the role of mitochondrial quality control (MQC) in B cells has not been thoroughly summarized, unlike in T cells. This article aims to review the mechanism of MQC that shapes B cell fate and functions. In addition, we will discuss the physiological and pathological implications of MQC in B cells, providing new insights into potential therapeutic targets for diseases associated with B cell abnormalities.

## 1. Introduction

Mitochondria are crucial organelles that orchestrate cellular energy metabolism and perform various functions, such as acting as the primary site of the tricarboxylic acid (TCA) cycle in eukaryotic cells, generating adenosine triphosphate (ATP) through oxidative phosphorylation (OXPHOS), regulating calcium homeostasis, and coordinating intracellular signal transduction [1]. It modifies their morphology and mass to adapt to different metabolic status or environmental stresses through the mitochondrial quality control (MQC) network, which helps organize these traits and maintain their proper function [2]. MQC encompasses mitochondrial biogenesis, mitochondrial dynamics (fission and fusion), mitophagy, and mitochondrial transport. These mechanisms ensure the regulation of the number, size, and location of mitochondria to maintain mitochondrial homeostasis [3]. Compromising the MQC systems can result in a range of diseases related to mitochondrial dysfunction, such as autoimmune diseases, neurological disorders, and cancer [4, 5].

Increasing evidence has elucidated the pivotal role of mitochondria in the immune system [6]. Mitochondria possess their own DNA that can be extracellularly released as web-like structures by immune cells [7]. Mitochondrial DNA (mtDNA) harbors a large amount of CpG islands that serve as damage-associated molecular patterns (DAMPs). This triggers the production of inflammatory cytokines via the NF-*κ*B pathway [8]. The mtDNA synergizes with mitochondrial reactive oxygen species (mtROS) to activate NLRP3 inflammasome, which then causes pyroptotic cell death [9]. In response to viral stimuli, mitochondrial antiviral signaling (MAVS) proteins, located on the outer mitochondrial membrane (OMM), activate NF-*κ*B and IRF3 signaling, inducing type-I interferon (IFN-I) in innate immune cells [10]. In instances relating to adaptive immune cells, mitochondria translocate to the immune synapse (IS) so that they can concentrate ATP production and calcium signaling to regulate T cell polarization and migration after activation. Mitochondrial metabolism is also responsible for regulating T cell proliferation and differentiation [11].

B cell-mediated adaptive immune response involves the production of antibodies, antigen presentation, and cytokine secretion, whose function is also determined by mitochondria [12]. B cell development originates from pluripotent hematopoietic stem cells (HSCs) found in the bone marrow. These cells further differentiate into common lymphoid progenitors (CLP). Progenitor B cells (pro-B cell), derived from CLP, begin to express Ig*α*/Ig*β* heterodimers and undergo V (D)J recombination. Pro-B cells continue to differentiate into large pre-B cells that express pre-B cell receptor (pre-BCR) [13]. Rapid proliferation and clonal expansion of large pre-B cells are accompanied by increased metabolic activity and mtROS production [14]. These large pre-B cells further develop into relatively quiescent small pre-B cells that complete light chain rearrangements. The rearrangements of heavy and light chain genes cause B cells to give rise to immature B cells that express the mature BCR (mIgM). At this point, if the BCR binds to autoantibodies in the bone marrow, B cells will lose self-reactivity and attain autoimmune tolerance through negative selection, including clonal deletion, receptor editing, and anergy [15]. Newly formed B cells migrate to secondary lymphoid organs. They circulate in the body via the blood and lymph. As they pass through transient transitional (T1 and T2/3) B cell stages, these cells gradually differentiate into naïve cells, mature follicular B (FoB) cells, or marginal zone B (MZB) cells [16, 17]. FoB and MZB cells represent two distinct populations of mature B lymphocytes with unique immune functions and anatomical locations. Follicular helper T (Tfh) cells interact with FoB cells in lymphoid organ follicles, leading to the emergence of germinal center B (GCB) cells that undergo class-switch recombination (CSR) and somatic hypermutation (SHM). These cells differentiate into long-lived plasma cells that generate high-affinity antibodies and memory B (Bm) cells [18]. The mouse MZB cells, located in the marginal zone of the spleen and responsible for the first line of defense against blood-borne pathogens, divide into short-lived plasma cells that rapidly produce low-affinity IgM antibodies [19]. It is notable that the human spleen lacks marginal sinus, which separates the marginal zone. Human MZB cells surround B cell follicles and circulate in the bloodstream [20–22]. In the past, MZB cells in human blood were regarded as Bm cells due to their expression of memory marker CD27 [23]. However, recent studies have shown that MZB and Bm cells occupy different microanatomical locations and remain largely clonally separate [24].

In the past 5 years, research in the field of B cells has aimed to uncover the distinct metabolic states of different cellular subsets and the impact of immune metabolism on cellular function [25, 26]. However, much less is known about mitochondria as a hub of cellular metabolism, which dynamically regulates metabolic signaling pathways such as OXPHOS and glycolysis and thus influences cell proliferation and division. Currently, research on B cell mitochondria lags behind that of T cells, and there are still many untouched aspects like the role of MQC in B cells. In this review, we highlight advances that mitochondrial biogenesis, fission and fusion, mitophagy, and mtROS in the control of B cell differentiation, activation, and immune response. We also discuss how changes in mitochondria may alter B cell proliferation and function, leading to pathological modifications.

## 2. Regulation of Mitochondrial Biogenesis and Its Relevance to B Cell Development, Activation, and Differentiation

Mitochondrial biogenesis is the process of increasing cellular mitochondrial mass, which occurs in response to capacity demands imposed by growth signals and environmental stresses. It was initially described in the 1960s by John Holloszy, who made the groundbreaking discovery that engaging in endurance exercise leads to an augmentation in the number of mitochondria present within skeletal muscles. This increase ultimately enhances the production of ATP and subsequently boosts one's capacity for enduring physical activity [27]. Mitochondria are semi-self-replicating organelles with their own genome. The mtDNA only encodes 13 essential components of the OXPHOS system, whereas the nucleus encodes more than 1000 proteins residing in mitochondria. This implies that mitochondrial biogenesis requires the cooperation of mtDNA and nuclear genes. It maintains intracellular homeostasis by regulating the distribution, mass, and mitochondria activity as well as the mtDNA replication [28].

### 2.1. Mitochondrial Biogenesis Machinery and Related Signaling Pathway

The process of mitochondrial biogenesis involves a complex transcriptional network that coordinates the transcription and replication of both the nuclear and mitochondrial genomes. The family of peroxisome proliferator-activated receptor *γ*-coactivator-1 (PGC-1), which includes PGC-1*α*, PGC-1*β*, and PGC-1, is responsible for the initial activation of mtDNA transcription. The nuclear respiratory factor-1 (NRF-1), NRF-2, and estrogen-related receptor-*α* (ERR-*α*) are subsequently activated with the activation of PGC-1*α* by either deacetylation or phosphorylation, ultimately leading to an increase in the expression of mitochondrial transcription factor A (TFAM). Additionally, TFAM also plays a crucial part in the replication of mtDNA, inducing a sharp bend into DNA and allowing the recruitment of RNA polymerase mitochondrial (POLRMT), which is required to form an RNA primer [29, 30]. Therefore, the PGC-1*α*-NRF-1/2-TFAM pathway orchestrates mitochondrial biogenesis.

### 2.2. Mitochondrial Biogenesis and Bioenergetics Collaborate to Control B Cell Development

Deletion of TFAM in B cells causes a developmental halt from pro-B cell to pre-B cell stage. The expression of most electron transport chain (ETC) proteins starts to rise gradually at the pre–pro-B stage and peaks in the earliest pre-B period. The loss of TFAM results in the transcription failure of mitochondrial encoding ETC-related genes, while the nuclear-encoded proteins are upregulated as a compensatory mechanism [31]. In addition, the rapid proliferation of the large pre-B cells requires large amounts of mitochondrial ATP production and increases glucose uptake. Large pre-B cells also product increased ROS through a highly active mitochondrial respiratory chain [32]. During the development of pre-B cells into small pre-B cells, the metabolic activity gradually shifts from a more active state to a more quiescent state, accompanied by a reduction in glycolysis [33]. Swiprosin-2/EFhd1 (EFhd1), a Ca^2+^-binding protein on the mitochondrial inner membrane, involved in mitochondrial bioenergetic events (mitoflashes) that maintain the proton motive force necessary for the generation of generate mitochondrial ATP in pro- and early pre-B cells. The expression of EFhd1 in pre-B cells was downregulated by the strength of pre-BCR, and its negative effect on glycolysis ultimately leads to a metabolic quiescence in small pre-B cells as well as late B cells [32, 33]. Despite the coexpression of EFhd1 with PGC-1*α*, a key gene for mitochondrial biogenesis, there was no significant difference in mitochondrial mass that was observed pro-B, large pre-B, and small pre-B cells [32]. Liculin-interacting protein 1 (Fnip1) is a critical factor in the development of B cells and the maintenance of metabolic balance. Fnip1-deficient pre-B cells exhibit aberrant cell growth due to uncontrolled mTOR activity, resulting in cell death. The increased mitochondrial biogenesis in these cells is attributable to AMP-activated protein kinase (AMPK) overactivation, which attempts to compensate for the loss of Fnip1, thereby failing to restore control over mTOR activity. This suggests that Fnip1 plays an indispensable role in regulating mTOR activity [34].

### 2.3. Increased Mitochondrial Mass During B Cell Activation, Regulation of Entry Into the GC, and CSR

Increased mitochondrial mass results from stimulation of B cell activation with lipopolysaccharide (LPS) for 6 h or anti-CD40 antibody with interleukin-4 (IL-4) for 24 h [35–37]. It might be necessary to meet the requirements of glycolysis and OXPHOS during early B cell activation. Upon encountering cognate antigens and receiving costimulation from helper T (Th) cells, naïve B cells undergo activation and subsequently migrate to secondary lymphoid tissues. After that, the activated antigen-specific B cells differentiate into GCB cells in GC where they undergo rapid expansion and proliferation [38, 39]. The microenvironment of GC is characterized as a site of intense cell proliferation with poor vascularization [40]. Compared to FoB cells, GCB cells have higher protein content, enhanced glucose uptake, and increased mitochondrial mass. As GCB cells experience high levels of proliferative stress, their demand for energy and nutrients increases to facilitate biosynthesis [41]. Glycogen synthase kinase 3 (Gsk3), a ubiquitously expressed kinase, acts as a metabolic sensor that constrains GCB cell growth and proliferation. Gsk3 facilitates c-Myc degradation by phosphorylating the transcription factor c-Myc [42]. The enhanced proliferation and metabolic activity of Gsk3-deficient B cells may be attributed to the increased expression of c-Myc, a protein that promotes mitochondrial biogenesis [41, 43]. TFAM, an important regulatory transcription factor for mitochondrial biogenesis, is required for the entry of activated GC precursor B cells into the GC program. In the absence of TFAM, the GC exhibits a markedly disorganized architecture, which results in a reduction in the generation of GCB cells, while a proportional increase in activated GC precursor B cells with population remains relatively stable. GCB cell precursors express Aicda but maintain immature markers. Therefore, TFAM is essential for the entry of GC precursor B cells into GC reaction [31]. At the later stages of GCB cell differentiation, cell clusters with high mitochondrial mass are preferentially subjected to CSR, yet those with low mitochondrial mass differentiate into plasma cells. Plasma cells have reduced mitochondrial mass, accompanied by an increased endoplasmic reticulum (ER) network [12, 37]. This phenomenon is possibly attributed to the production and secretion of immunoglobulins by plasma cells, which causes an increase in ER stress [44].

## 3. Mitochondrial Dynamics Dictate the Fate and Function of B Cells

In contrast to isolated static organelles, mitochondria are highly dynamic. In order to adapt to the energy demands of cell, mitochondria changes in their shape, size, and position through fission, fusion, and transport [45, 46]. During mitochondrial fission, small individual mitochondria are generated, and the damaged mitochondria are separated and distributed to daughter cells during cell division. However, mitochondrial fusion is the process of merging two organelles into one, allowing their contents to be exchanged between different mitochondria and preventing mitochondria degradation [4, 47].

### 3.1. Molecular Machinery of Mitochondrial Dynamics: Fusion and Fission

The cytosolic GTPase dynamin-related protein 1 (Drp1) controls the fission of mitochondria [48, 49]. Fission protein 1 (FIS1), mitochondrial elongation factor 2 (MIEF2/MID49), mitochondrial fission factor (MFF), and mitochondrial elongation factor 1 (MIEF1/MID51) are adaptor proteins that facilitate the translocation of Drp1 from the cytosolic pool to the OMM [50–52]. Drp1 self-assembles a ring-like structure that wraps around and constricts mitochondrial tubules before pinching the mitochondrion apart [53]. Mitochondrial fusion requires the presence of three dynamin family GTPases, mitofusin 1 and 2 (Mfn1/2), and optic atrophy protein 1 (Opa1). Mfn1 and Mfn2, located on the OMM, are responsible for OMM fusion [54]. The inner mitochondrial membrane (IMM) fusion is regulated by Opa1 [55, 56]. Opa1 is available in two distinct forms: long forms (L-Opa1) and short forms (S-Opa1). L-Opa1 can be proteolytically cleaved by two proteolytic enzymes named OMA1 and YME1L1 in the IMM to generate S-Opa1 [57]. The functions of the two forms of Opa1 are different. L-Opa1 is mainly responsible for IMM fusion, while S-Opa1 induces mitochondrial fission [57]. In addition to regulating mitochondrial fission and fusion, Opa1 also dynamically controls cristae morphology, OXPHOS supercomplex assembly, and mtDNA maintenance [58]. T cell subsets, such as T helper 17 (Th17) cells and memory T (Tm) cells, have fused mitochondria with tight cristae. Conversely, T effector (Teff) cells undergo fission with smaller and dispersed mitochondria. Due to the disruption of mitochondrial cristae and decreased OXPHOS activity, the differentiation of Th17 and Tm cells is impaired in the absence of Opa1 [59, 60].

### 3.2. The Role of Mitochondrial Dynamics and Metabolic Reprogramming in B Cell Fate and Function

**Figure 1:**
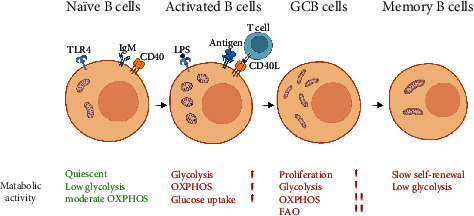
Mitochondrial dynamics in B cell activation. Naïve B cells are in a relatively quiescent state and rely on the mitochondria to maintain cellular metabolism. Activation of B cells leads to elevated levels of glycolysis accompanied by increased mitochondrial mass. Following entry into the germinal center, germinal center B (GCB) cells are inclined toward fatty acid oxidation (FAO) for oxidative phosphorylation (OXPHOS), and the mitochondria exhibit a fused state with a tighter mitochondrial cristae formation. Memory B cells exhibit fused mitochondria, similar to memory T cells.

Similar to T cells, B cells modify mitochondrial dynamics and morphology to adjust to the energy demands of various differentiation stages. B cells activated by CD40/IL-4 in vitro exhibit more numerous, smaller, and rounder mitochondria compared to those of naïve B cells. However, the genomic DNA and mtDNA replication in activated B cells remained at the same level as in naïve B cells. In activated B cells, the number of nucleoids per mitochondria decreased, while the total nucleoid area increased. It suggests while CD40/IL-4 activated B cells, mitochondria undergo fission to generate more mitochondria without replicating their mtDNA through mitochondrial biogenesis [36]. In contrast, sheep red blood cells (SRBCs) induced GCB cells from immunized mice possessed larger fused mitochondria than those of naïve B cells [31]. Mitochondrial fission and fusion are accompanied by changes in energy metabolism. Mitochondrial fusion facilitates the entry of pyruvate into the mitochondria for oxidation rather than the secretion of lactic acid, while fatty acid oxidation (FAO) may be the default pathway of the mitochondrial fusion state [59]. Recent studies have demonstrated that GCB cells tend to be FAO dependent, with minimal glycolysis [61], which contrasts with the metabolic state observed in proliferating T and B cells. Furthermore, GCB cells predominantly rely on OXPHOS rather than aerobic glycolysis, and post-GCB cells require OXPHOS during their differentiation into plasmablasts [62, 63]. Typically, longer fused mitochondria maintain tight mitochondrial cristae and are associated with increased OXPHOS, while fragmented mitochondria have less OXPHOS capacity [64]. When mitochondria stay fused, mtDNA in the matrix, and IMM respiratory proteins are able to mix between the mitochondria, which facilitates the maintenance of mitochondrial respiratory strands and mtDNA integrity, so as to ensure efficient and effective cellular OXPHOS [65–67]. Therefore, the fused mitochondria of GCB cells are probably fulfilling the metabolic requirements of FAO and OXPHOS. Moreover, mitochondrial fusion also promotes the differentiation of GCB to Bm cells and plasma cells. Mdivi-1, an inhibitor of mitochondrial fission, promoted the production of Bm cells and enhanced mitochondrial function in immunized mice. Further, Mdivi-1 could be used as an “immune booster” to increase the effectiveness of vaccines [68]. Bm cells may benefit from more fused mitochondria for catabolic metabolism and long-term survival. Therefore, Bm cells may contain elongated mitochondria morphology similar to Tm cells [59], although their specific pattern of mitochondrial morphology needs to be further demonstrated in more extensive research (Figure 1).

The biological function of B cells can be regulated by tumor necrosis factor receptor-associated factor 3 (TRAF3), a cytoplasmic adaptor protein of the TRAF family that is predominantly located in the mitochondria and interacts with MFF [69]. MFF is a key adaptor protein in the OMM to promote mitochondrial division [70]. In the absence of survival factors, Traf3^−/−^ B cells contained healthy and elongated morphology of mitochondria after 1 day of culture in vitro, whereas normal B cells exhibited abnormally irregular vacuoles and a loss of mitochondrial cristae [71]. Interestingly, B cell-specific knockdown of Traf3 resulted in improved B cell survival, enhanced BCR and toll-like receptor (TLR) signaling, and promoted plasma cell differentiation [69, 72–75]. It is inferred that fused mitochondria are essential for the survival and further differentiation of B cells.

## 4. Modulation of Mitophagy in B Cell

Autophagy is the process through which lysosomes degrade cytoplasmic proteins and damaged organelles [76]. Mitophagy, also known as mitochondrial autophagy, is a form of macroautophagy that selectively separates mitochondria and delivers them to lysosomes for degradation [77]. Mitochondria are susceptible to damage when cells are subjected to oxidative and metabolic stresses [78, 79]. In order to maintain mitochondrial and cellular homeostasis and prevent damaged mitochondria from injuring cells, mitophagy can effectively eliminate dysfunctional mitochondria.

### 4.1. Molecular Machinery of Mitophagy

The mechanisms of mitophagy mainly rely on ubiquitin (Ub)-dependent or Ub-independent (receptor dependent) initiating pathways [80, 81]. Ub-dependent pathways are primarily regulated by the PTEN induced putative kinase 1 (PINK1)/Parkin pathway [82]. PINK1, a serine/threonine kinase, is imported from the OMM to the IMM and degraded by several proteases under physiological conditions [83]. When mitochondrial membrane potential (*ΔΨ*m) is lost, Pink1 cannot enter the IMM and instead accumulates on the OMM surface. On the OMM surface, Pink1 recruits and phosphorylates Parkin [84]. E3 Ub ligase activity of Parkin is activated, resulting in the degradation of a variety of OMM proteins by ubiquitination [83]. These ubiquitinated OMM proteins interact with LC3 to form autophagosome [85]. Several receptors in OMM, like Nip3-like protein X (NIX), FUN14 domain containing 1 (FUNDC1), and BCL2-interacting protein 3 (BNIP3), can directly bind LC3 without interacting with Parkin [86–89]. Thus, mitophagy depends on PINK1/Parkin pathway and membrane surface receptors to remove dysfunctional mitochondria.

### 4.2. Mitophagy Controls the Formation of Bm Cells

Regardless of whether B cells are stimulated with BCR or TLR signaling, autophagy is always associated with their activation [44, 90]. Of note, NP-CGG-immunized Atg5^f/f^CD19-Cre mice exhibited a GC formation comparable to that observed in their control Atg5^f/f^ littermates, as assessed by immunohistochemistry and flow cytometry. Additionally, when analyzing sorted CD19^hi^FAS^hi^CD38^lo^ GCB cells, no preferential selection for nonrecombinant loxP-flanked Atg5 was observed. Thus, the absence of the classic autophagy gene ATG5 still has a normal GC response; autophagy was dispensable for GC formation [44]. Instead, GCB cells rely more on nonclassical autophagy flux regulated by phosphoinositide-interacting protein 2 (WIPI2) [91, 92]. Differently, B cells deficient in the autophagy genes ATG7 or ATG5 damaged plasma cell differentiation [44, 93] and the long-term persistence of Bm cells [94]. Autophagy facilitates the scavenging of damaged mitochondria and prevents ER stress during B cell activation [93]. Mitophagy, as a specific form of autophagy, also prevents the accumulation of damaged mitochondria and acts as an essential mechanism for the survival of Bm cells. Compared to GCB cells, Bm cells have a reduced mitochondrial content. Bm cells deficient in both the mitochondrial autophagy genes Nix and Binp3 have impaired mitophagy and contain more active mitochondria, leading to reduced generation and a failure to survive long-term [95]. Nix^−/−^Bnip3^−/−^ Bm cells show elevated OXPHOS capacity and de novo fatty acid synthesis which induce necroptosis signaling [95]. Interestingly, the metabolic capacity of Nix^−/−^Bnip3^−/−^ Bm cells is rescued by treatment with metformin, which leads to the activation of AMPK [95]. AMPK, a key regulator of autophagy and metabolism, enhances the long-term persistence and function of Bm cells by promoting mitochondrial clearance and prevents cellular lipid peroxidation [96]. AMPK also restricts plasma cells from producing excessive amounts of antibodies [96]. A recent study has shown that p66SHC limits the differentiation of plasma cells by activating the AMPK and LC3-II to promote mitophagy [97]. Collectively, mitophagy effectively clears superfluous mitochondria in Bm cells to protect them from excessive oxidative stress and cell death. However, p66SHC-dependent mitophagy restricts B cell differentiation to plasma cells.

In addition, mitochondrial inner membrane proteins are found to regulate mitophagy; abnormal function of these proteins may lead to disorders of mitophagy, thereby affecting B cell survival and maturation. ATPase family AAA domain-containing protein 3A (ATAD3A), an IMM protein, promotes the translocation of PINK1 to the IMM for processing and inhibits the expression of Pink1, preventing mitophagy [98]. A mouse model of hyperactivated mitophagy induced by deleting ATAD3A in HSCs and committed progenitor cells reveals a failure of progression from pre–pro- to pro-B cell stage and decreased frequency of B220^+^ B cells in the peripheral blood [98].

## 5. mtROS Are Signal Regulators in B Cell

ROS have a dual nature. On one hand, they are toxic substances that can damage cellular DNA, proteins, and lipids. On the other hand, they are essential for maintaining cellular physiological function [99, 100]. Intracellular ROS are prominently derived from the mitochondrial ETC and nicotinamide adenine dinucleotide phosphate (NADPH) oxidases [101]. ROS can also be produced via 5-lipoxygenase (5-LO) pathway in CD40-stimulated B cells [102]. mtROS, act as by-products of OXPHOS, closely associated with mitochondrial dynamics. In general, fragmented mitochondria induced more mtROS production, whereas elongated mitochondria had a lower production of mtROS [103, 104]. ROS also alters mitochondrial morphology. High levels of ROS promote mitochondrial fission, while low ROS favor mitochondrial fusion [105–107]. In addition, mitophagy limits excess ROS production after oxidative damage by removing damaged mitochondria [108]. mtROS production is required for appropriate immune response. T cells produce mtROS during activation. mtROS signaling promotes antigen-specific amplification and supports interleukin-2 (IL-2) induction [109].

**Figure 2:**
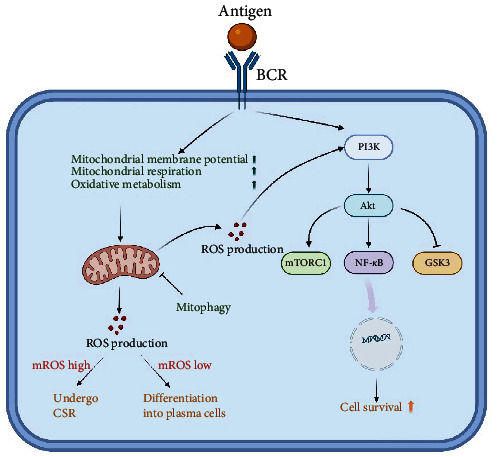
Mitochondrial reactive oxygen species (mtROS) determine B cell activation and plasma cell formation. Upon antigenic stimulation, B cell receptor (BCR) signaling is activated, leading to an increase in oxidative metabolism, mitochondrial membrane potential, and mitochondrial respiration in B cells. This process results in an increase in mtROS levels during the late stage of activation, which enhances activated PI3K signaling and promotes cell survival. High levels of mtROS promote B cells to undergo class-switch recombination (CSR), while low levels of ROS promote plasma cell differentiation.

ROS influences the signaling of multiple immune cell receptors, which include BCR, TCR, TLR, and cytokine receptors [110]. ROS is considered a second messenger for BCR signaling. The ROS production is transient and modest in the initial phase following BCR stimulation, which does not regulate B cell function. Nevertheless, heightened and prolonged levels of ROS are produced in the advanced phases of BCR stimulation, contributing to the proliferation and activation of B cells. Controversially, some researchers have proposed that NADPH oxidases are the source of ROS production in the late phase [111], while others have suggested that it predominantly leaks out of the mitochondrial respiratory chain [112]. The reasons for this discrepancy remain elusive. However, B cells exhibit a preference for relying on mitochondrial oxidative metabolism in response to T cell-dependent stimulation, leading to elevated *ΔΨ*m and mtROS production [113]. This proposes that the mitochondrial respiratory chain might be a significant source of ROS after BCR stimulation. Boosted mtROS production is induced by upregulated mitochondrial activity and mitochondrial respiration in the GSDMA3 KO B cells. GSDMA3, a member of the gasdermins (GSDM) subfamily, has been identified as regulating mitochondrial oxidative stress [114]. In the absence of GSDMA3, B cells experience impaired BCR signaling, along with decreased BCR clustering and BCR signalosome recruitment. Nevertheless, B cell physiological activities are not affected by GSDMA3 deficiency [115]. The unaffected B cell function may be due to the fact that mtROS promotes PI3K signaling [116], which acts as a downstream of BCR signaling to compensate for the impairment of BCR signaling. A recent study has demonstrated that low levels of intracellular ROS can promote the differentiation of B cells into plasmablasts [117]. These plasma cells show higher oxidative metabolism to secrete antibodies [63]. As a consequence of OXPHOS, plasma cells exhibit higher levels of ROS generated by mitochondria compared to naïve B and GCB cells. Accordingly, Jang et al. have shown that the differentiation of plasma cells relies on appropriate levels of mtROS, although high levels of mtROS lead to preferential CSR. Elevated mtROS levels impede plasma cell differentiation by attenuating haeme synthesis which inhibits Bach2 function and thus de-represses Blimp1, a master regulator of plasma cells [37]. Nonetheless, when a sudden or sustained rise in mtROS formation that is not efficiently decomposed and eliminated, irreversible and deleterious damage will occur in B cells [118, 119]. Thus, physiological levels of mtROS produced by normal metabolic requirements are critical for the maintenance of BCR signaling as well as B cell differentiation and development (Figure 2).

## 6. Failure of MQC Contribute to B Cell Lymphoma

B cell lymphoma is the most prevalent kind of lymphoma, accounting for approximately 70% of all cases around the world [120]. Abnormal V (D)J rearrangements, SHM, and CSR lead to altered B cell survival and proliferation, resulting in B cell lymphoma [121]. B cells at different stages of differentiation can undergo malignant transformation while retaining primary features of their cellular origin and subsequently develop into lymphomas [122]. For example, the most prevalent form of lymphoma is diffuse large B cell lymphoma (DLBCL), which originates from GCB and activated B cells. DLBCL is accompanied by three major antigen receptor gene rearrangements, including MYC, BCL2, and BCL6 [123]. In cases of DLBCL, the overexpression of MYC and BCL2 is associated with a high risk of disease relapse and indicates a poor prognosis [124]. MYC inhibits mitophagy-mediated necroptosis to promote disease progression in DBCL [125]. Interestingly, the knockdown of TFAM reversed the carcinogenic effects of c-MYC. Deletion of TFAM can prevent the development of B cell lymphoma by inhibiting mitochondrial translation [31]. It seems that mitochondrial biogenesis-related genes contribute significantly to the development of B cell lymphoma and hold promise as potential therapeutic targets.

Hodgkin's lymphoma and non-Hodgkin's lymphoma (NHL) are two types of B cell lymphoma. The risk of NHL appears to be associated with mitochondrial dysfunction. mtDNA are more susceptible to oxidative damage due to its lack of introns and protective histones compared to nuclear DNA. When mitochondria perceive excessive oxidative damage, an increased mtDNA copy number substitutes limited DNA repair capacity, ultimately resulting in increased release of proinflammatory cytokines [126, 127]. Recent clinical studies have shown that the risk of developing NHL is associated with mtDNA fraction breaks and increased mtDNA copy number [128, 129]. It follows that the level of mtDNA may be associated with NHL risk.

Impairment of mitophagy also promotes B cell lymphoma progression. Oncogenic MYC impedes necroptosis in B lymphoma cells by inhibiting mitophagy through enhanced expression of the key enzyme phosphate cytidylyltransferase 1 choline-*α* (PCYT1A). Silencing of the PCYT1A in B lymphoma cells displayed significant necroptosis induction and growth inhibition. When treated with mitophagy inhibitor Mdivi-1, necroptosis was reversed [125]. On the other hand, the activation of PINK1/Parkin-mediated mitophagy by ZnO nanoparticles in DLBCL cells effectively inhibited tumor cell growth. ZnO nanoparticles also induce massive mtROS production, which disrupts intracellular physiological homeostasis and ultimately leads to cell death [130]. It has also been found that B cell lymphoma cells exhibit reduced growth and increased mtROS levels when treated with mitochondrial transcription and translation suppressors [31]. Targeting mitophagy will prospectively be an effective treatment for B cell lymphoma.

## 7. Mitochondria and Metabolic Dysfunction in B Cell-Mediated Autoimmune Diseases

A breach of autoimmune tolerance, which results in the destruction of normal cells and histopathological damage, characterizes autoimmune diseases [131]. B cells are now recognized as a significant mediator of autoimmune etiology due to the clinical success of B cell deletion therapies, utilizing anti-CD20 antibodies in disorders such as systemic lupus erythematosus (SLE), rheumatoid arthritis (RA), and multiple sclerosis (MS) [132]. Both animal models of autoimmunity and patients suffering from autoimmune diseases exhibit an expansion of self-reactive B cells. These autoreactive B cells are essential in the development of autoimmune diseases, including autoantibody secretion, antigen presentation and processing, proinflammatory cytokine secretion, and the formation of ectopic lymphoid follicular tissue [133].

In many autoimmune diseases, a biomarker is the hyperactivation of B cells, which requires a high level of cellular energy generated mainly by the mitochondria [134]. B cell-activating factor (BAFF), a well-known tolerance checkpoint, is significantly elevated in the serum of autoimmune disease patients, and its neutralizing antibody, belimumab, is a widely used biologic agent for the treatment of lupus nephritis [135–138]. Notably, imbalances in glycolysis and OXPHOS metabolism may allow autoreactive B cells to evade self-tolerance checkpoints. B cells chronically exposed to high levels of BAFF demonstrate an increase in mitochondrial capacity and elevated levels of glycolysis and OXPHOS upon stimulation. Elevated mitochondrial membrane potential is caused by excessive BAFF signaling, which also promotes pyruvate influx into mitochondria for oxidative metabolism [35, 139]. The alternative NF-*κ*B signaling pathway is triggered by BAFF receptor which binds with its ligand; this pathway is characterized by NF-*κ*B-inducing kinase (NIK) initially activated and subsequently phosphorylates and activates IKK*α*, which in turn induces the proteasome-mediated processing of the precursor protein p100 into the mature NF-*κ*B2 p52 [140]. NIK which is localized to the mitochondria promotes mitochondrial fission by enhancing the recruitment of Drp1 [141]. In naïve B cells, NIK can form a complex with TRAF3. TRAF3 suppresses the activity of the alternative NF-*κ*B pathway by inhibiting NIK [142]. Once BAFF signaling is engaged, TRAF3 is degraded, and alternative NF-*κ*B signaling is activated. Subsequently, stabilized NIK enhances Drp1 recruitment and promotes mitochondrial fission. B cell-specific deficiency of TRAF3 increases spontaneous GC formation and the risk of autoimmunity, which may be explained by enhanced B cell survival through altering mitochondrial morphology and energy metabolism [71, 75, 143]. Thus, mitochondria are crucial for maintaining the survival of autoreactive B cells, which boost metabolic capacity by enhancing mitochondrial fission. In addition, autoimmune disorders are prone to oxidative damage, which leads to increased release of mtROS into the cytosol [144]. The generation of proinflammatory cytokine may be directly influenced by mtROS [145]. These proinflammatory cytokines, such as TNF-*α* and IL-6, may be involved in local or even systemic inflammatory responses that exacerbate the progression of autoimmune disease [146].

Traditionally, metformin was used to treat type 2 diabetes by lowering blood glucose levels. Recent studies have shown that metformin reduces glomerular IgG and complement C3 deposition as well as downregulates proinflammatory cytokines such as MCP-1, TNF-*α*, and IL-1*β* to achieve remission of the pathological process of SLE [147]. Metformin also significantly suppresses the formation of GCB cells and the differentiation into antibody-secreting plasma cells in SLE [148]. Mechanically, metformin may inhibit GCB cells expansion by reducing intracellular ATP and ROS production through inhibition of mitochondrial respiration and OXPHOS [149]. In addition, metformin effectively inhibited BAFF-induced proliferation and survival of B cells [150]. Hence, targeting mitochondria to regulate B cell metabolism and thereby preventing the formation of autoreactive cells will be an effective treatment for autoimmune diseases.

A wide number of studies have shown that targeting mitochondria controls T cell differentiation and function in autoimmune diseases [151]. However, there is still a significant vacuum in the understanding of B cell mitochondria in autoimmune diseases. Targeting mitochondria to regulate metabolism has the potential to modulate the production of proinflammatory cytokines and antibody secretion in autoreactive B cells.

## 8. Conclusion and Future Perspective

In this review, we summarize recent findings on the regulation of B cell metabolism and biological functions through mitochondrial mass, morphology, mitophagy, and mROS. Dysregulation of mitochondrial dynamics, cellular metabolic status, and biological activity can lead to severe imbalances in subpopulation ratios, leading to B cell lymphoma, autoimmune diseases, and other related disorders (Table 1). Mitochondria play a crucial role as a powerhouse for immune cells. Research in the last decade has shed light on the unique mitochondrial metabolism, mass, and morphology of different immune cell subsets, especially T cells. Mitochondrial morphology and metabolism differ significantly between B and T cells. For example, rapidly proliferating effector T cells perform mitochondrial fission and primarily undergo glycolysis, the fermentation of glucose to lactate, rather than oxidation in the mitochondria [59]. However, recent studies have shown that proliferating GCB cells are more dependent on mitochondria for OXPHOS using fatty acids with minimal glycolysis [61]. B cells distinguish themselves from other immune cells by having unique mitochondrial mass control to support different energy changes during differentiation and activation. Many questions remain regarding the mechanisms of mitochondrial dynamic change in B cells. Future studies should aim to uncover the unique mitochondrial dynamics in different B cell subsets in physiological and pathological states, how these highly variable mitochondria coordinate mitochondrial metabolic pathways to influence B cell development and biological functions, and how mitochondrial transcription factors alter metabolic states to affect cell fate and function. The advancement of electron microscopy, single-cell sequencing, and spatial metabolome technologies enables the exploration of the relationship between alterations in mitochondrial dynamics and metabolism within specific B cell subpopulations. These studies will deepen our understanding of how mitochondrial dynamics regulate B cell function and may lead to the development of new therapeutic strategies for immune-related disorders.

## Figures and Tables

**Table 1:** Summary of the relationship between mitochondrial quality control and B cell biology.

B cell subpopulation	Mitochondrial quality control features	Mitochondrial quality control-related genes	Metabolic profile	Functional relationship between mitochondrial dynamics and metabolic state	Mitochondrial dysfunction in tumorigenesis or autoimmunity
Pro-B cell	High *ΔΨ*m	—	High glycolysis	—	—
Large pre-B cell	Increased mtROS production [32]	EFhd1 [32] PGC-1*α* [34] Tfam [31]	High glucose uptake	—	Lymphomagenesis [34]
Small pre-B cell	Lower *ΔΨ*m [32]	EFhd1 [32]	Downregulated glycolysis [32]	—	—
Naïve B cell	Low mitochondrial mass	—	Quiescent Low glycolysis Moderate OXPHOS	Mitochondria perform minimal OXPHOS energy supply	—
Activated B cell	Increased mitochondrial mass and fission [35, 36]	—	Increased glycolysis OXPHOS [36]	Mitochondrial fission for more efficient glycolysis and energy supplement	—
GCB	Favored mitochondrial fusion [31]	Gsk3 [41] Tfam [31]	Tendency to rely on OXPHOS and FAO [61, 62]	Fused mitochondria are more efficient for OXPHOS [64]	Lymphomagenesis [31]
Long-lived plasma cell (LLPC)	Dependent on moderate levels of mitochondrial ROS [37] Increased mitochondrial mass [152]	—	High glucose uptake and increased mitochondrial respiratory capacity [152]	Plasma cells are hypothesized to possess fused mitochondria for OXPHOS	—
Short-lived plasma cell (SLPC)	Equal mitochondria numbers to LLPC [153]	—	Tendency to rely on OXPHOS [63]	—	—
Bm	Reduced mitochondrial content Favored mitochondrial fusion like Tm	Nix, Bnip3 [95]	Quiescent	Mitophagy maintains mitochondrial self-renewal	—

Abbreviations: FAO, fatty acid oxidation; GCB, germinal center B; mtROS, mitochondrial reactive oxygen species; OXPHOS, oxidative phosphorylation; pro-B cell, progenitor B cell.
